# Is microRNA-33 an Appropriate Target in the Treatment of Atherosclerosis?

**DOI:** 10.3390/nu15040902

**Published:** 2023-02-10

**Authors:** Malgorzata Sidorkiewicz

**Affiliations:** Department of Medical Biochemistry, Faculty of Health Sciences, Medical University of Lodz, 90-419 Lodz, Poland; malgorzata.sidorkiewicz@umed.lodz.pl

**Keywords:** microRNA-33, HDL, atherosclerosis, cholesterol efflux, reverse cholesterol transport, bile acids

## Abstract

The maintenance of cholesterol homeostasis is a complicated process involving regulation of cholesterol synthesis, dietary uptake and bile acid synthesis and excretion. Reverse cholesterol transport, described as the transfer of cholesterol from non-hepatic cells, including foam cells in atherosclerotic plaques, to the liver and then its excretion in the feces is important part of this regulation. High-density lipoproteins are the key mediators of reverse cholesterol transport. On the other hand, microRNA-33 was identified as a key regulator of cholesterol homeostasis. Recent studies indicate the impact of microRNA-33 not only on cellular cholesterol efflux and HDL production but also on bile metabolism in the liver. As proper coordination of cholesterol metabolism is essential to human health, discussion of recent findings in this field may open new perspectives in the microRNA-dependent treatment of a cholesterol imbalance.

## 1. Introduction

**Figure 1:**
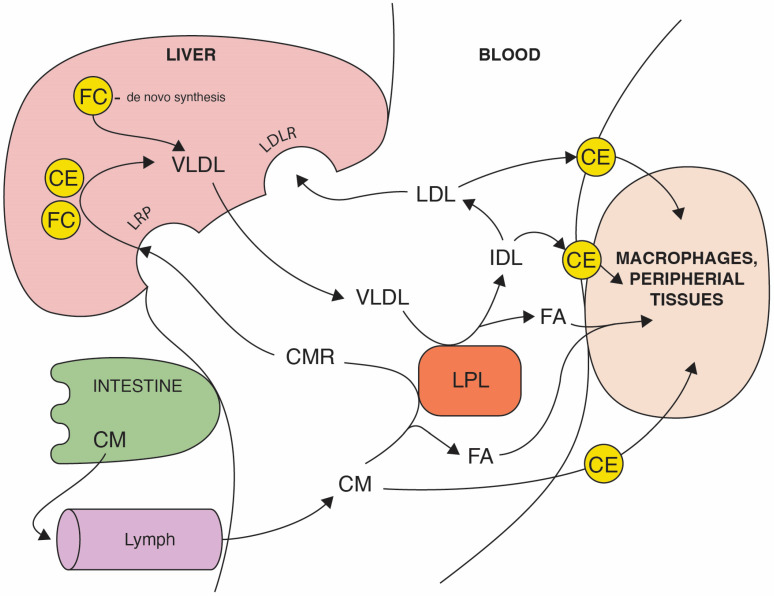
The route of dietary and endogenous cholesterol in an organism. The absorption of dietary triglycerides, free cholesterol (FC), and cholesterol esters (CE) occurs in the small intestine. In enterocytes, dietary lipids are packed in chylomicrons (CM) that are diffused to the bloodstream via lymph. After the digestion of triglycerides by lipoprotein lipase (LPL), fatty acids (FA) are absorbed by target cells and chylomicrons remnant (CMR) uptake to the liver is mediated by LDL receptor-related protein (LRP). The endogenous cholesterol pathway starts from the liver, recirculating triglycerides and cholesterol in the bloodstream, packed in very low-density lipoproteins (VLDL). VLDL through the LPL activity delivers FA to peripheral cells and are metabolized to intermediate-density lipoproteins (IDL) which are enriched in cholesterol. Low-density lipoproteins (LDL) derived from IDL carry the majority of the cholesterol and transports mostly esterified cholesterol (CE) to peripheral tissues, including macrophages in vein walls. LDL are eventually cleared by the liver via the LDL receptor.

The loss of cholesterol homeostasis is associated with major cardiometabolic risk factors including atherosclerotic cardiovascular disease and type 2 diabetes [1]. Cholesterol is an important structural component of biological membranes and a substrate for the synthesis of other steroids; thus, both cholesterol intake from the diet as well as its delivery from endogenous synthesis are essential for the maintenance of sufficient concentration of cholesterol in blood. On the other hand, aberrant cholesterol content is linked to coronary atherosclerosis and to other diseases [2,3]. Cholesterol synthesis de novo occurs in the human body through a series of enzymatic reactions from a commonly present substrate, acetyl-CoA. Additionally, cholesterol with triglycerides is delivered to cells from the circulation in the form of lipoproteins [4]. Blood lipoproteins are complex particles that contain cholesterol esters and triglycerides in a central core that is surrounded by free cholesterol, phospholipids and variety of apolipoproteins (Apo) that are responsible for lipoprotein formation and function. Two types of non-high-density lipoproteins, containing apolipoprotein apoB, are involved in lipid delivery to cells (Figure 1). The first one, chylomicrons (CL), equipped with apolipoprotein B-48, are produced from diet-delivered lipids in the intestine. The second, very low-density lipoproteins (VLDL), containing apolipoprotein B-100, are synthetized endogenously in the liver. In peripheral tissues, mostly in muscles and adipose tissue, triglycerides from CL and VLDL are broken down by lipoprotein lipase (LPL), releasing free fatty acids as well as chylomicrons remnants and intermediate-density lipoproteins (IDL), respectively. IDL are then metabolized to low-density lipoproteins (LDL) that are taken up via the LDL receptors both on the surface of both extrahepatic cells as well as on the surface of the liver, the predominant site of LDL uptake. A chronic inflammatory disease, atherosclerosis, is characterized mainly by the LDL-dependent deposition of excess cholesterol in the arterial walls [5]. Circulating LDL can transcytose across the endothelium and accumulate in the arterial wall, leading to the formation of atherosclerotic plaque [6]. The progression of the disease is associated with a number of different cells such as macrophages, endothelial cells and vascular smooth muscle cells [7].

**Figure 2:**
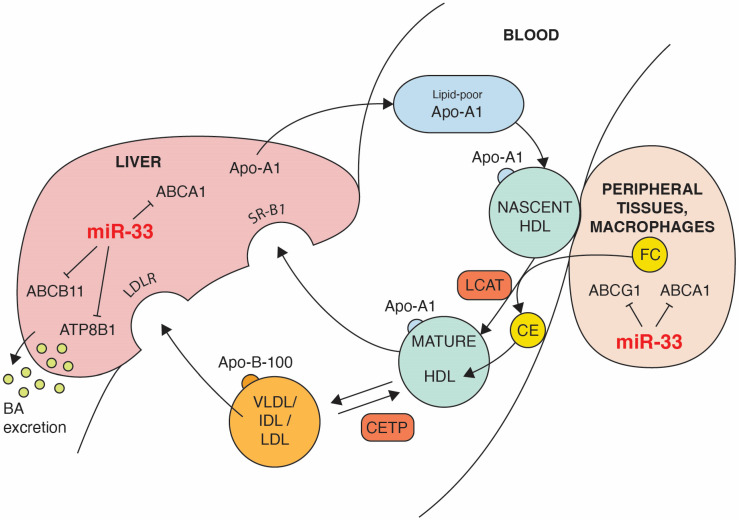
The impact of miR-33 on some steps of reverse cholesterol transport (RCT). Black lines ending with a short perpendicular line indicate target genes that are repressed by miR-33. ABCA1-mediated lipidation of lipid-poor apolipoprotein A-I (Apo-A1) forms nascent HDL. Cholesterol efflux from extrahepatic cells, including macrophages, is mediated by ABCA1 and ABCG1 activity. Through the action of the lecithin:cholesterol acyl transferase (LCAT), free cholesterol (FC) is esterified and cholesterol esters (CE) are absorbed inside of HDL. The progressive action of LCAT on nascent HDL generates a spectrum of mature HDL particles. Part of CE can be transferred by cholesterol ester transfer protein (CETP) from HDL to apolipoprotein B-100 containing lipoproteins (VLDL, IDL, LDL) in exchange for triglycerides (TG), promoting cholesterol clearance by the LDL receptor. Hepatic SR-BI mediates removal of FC and CE from HDL. Excess cholesterol is converted to bile acids excreted from the liver into the bile. Both the ABC11 and ATP8B1 transporters promote hepatic excretion of the bile.

An opposite action to LDL is characteristic of the high-density lipoproteins (HDL) fraction containing apolipoprotein A1 (Apo-A1). HDL, by stimulating the efflux of an excess of cellular cholesterol from extrahepatic tissues and transporting it back to the liver (Figure 2), plays a key role in the maintenance of cholesterol homeostasis [8]. The dyslipidemia characterized by an increased concentration of LDL and triglycerides and a decreased level of HDL remains a major factor of cardiovascular diseases [9]. It was only in the second half of the twentieth century when it became clear that HDL concentration and apo-A1 are inversely correlated with cardiovascular risk. This discovery has consequently led to the identification of the reverse cholesterol transport (RCT) pathway as essential for protection against atherosclerosis. RCT promotes cholesterol efflux from artery walls to HDL by activating a series of factors such as: transporters, enzymes and receptors (Figure 2). The ATP-binding membrane cassette transport proteins, ABCA1 and ABCG1, seem to be crucial for both HDL formation and the cholesterol efflux in RCT process [10,11]. Cholesterol homeostasis is closely controlled by feedback mechanisms, and an important part of it depends on the regulation of RCT. At the transcriptional level, the sterol-activated liver X-receptor (LXR) seems to play a key role in the regulation of RCT [12]. In vivo, LXR is activated by oxysterols in cholesterol-loaded cells, and it controls the response to cholesterol excess by regulating target genes *ABCA1* and *ABCG1.* Increased expression of ABCA1 and ABCG1 transporters [13,14] mediates cholesterol efflux in macrophages, reducing cellular sterol content.

The sterol regulatory element-binding proteins 1 and 2 (SREBP-1 and SREBP-2) are the next important factors controlling the transcription of genes involved in the regulation of cholesterol metabolism [15,16,17]. While SREBP-1 is induced by hyperinsulinemia and is mostly involved in the synthesis of fatty acids, SREBP-2 controls the intracellular cholesterol status [16]. If cells accumulate an excess of cholesterol or other sterols, inhibition of SREBP processing causes the decrease of LDL receptor expression, limiting the influx of cholesterol into the cell. At the same time, the activity of rate-limiting enzyme for *the novo* cholesterol synthesis, 3-hydroxy-3-methylglutaryl CoA reductase, declines. Both effects, the decrease of cholesterol uptake and the decline of intracellular cholesterol synthesis, are controlled by SREBP. Recently, microRNAs (miRNAs) were identified as important transcription regulators that modulate numerous cellular processes including the cholesterol homeostasis. Several lines of evidence indicate that microRNA-33 (miR-33) regulates cholesterol metabolism in cooperation with the SREBP host genes [18]. On one hand, miR-33 was demonstrated to reduce cholesterol efflux and RCT by targeting genes involved in cholesterol export [11]. On the other hand, it was demonstrated that the inhibition of miR-33 expression improves the blood cholesterol profile and promotes reversing of atheroma [19,20]. Thus, miR-33 appears to plays an important role in the complicated, multifactorial process of maintaining a balance between dietary cholesterol and blood cholesterol. Moreover, it suggests the potential of miR-33 inhibition as a therapeutic target in the treatment of dyslipidemia and atherosclerosis [21]. This review shows how miR-33 expression may have an influence on the homeostasis of cholesterol by indicating the impact of miR-33 on HDL biogenesis, RCT and bile acid synthesis.

## 2. How Can microRNA-33 Regulate Cholesterol Metabolism?

Careful coordination of cholesterol uptake, biosynthesis and use is essential for human health. That explains why cholesterol metabolism is tightly regulated at a cellular and whole-body level. In addition to the classical regulators of cholesterol expression, sterol regulatory element-binding proteins, numerous noncoding RNAs (including microRNAs) play an essential role in this process. The human genome contains thousands of non-coding RNAs. MicroRNAs are a class of short non-coding RNAs with 18–22 nucleotides [22] that regulate the expression of target genes as sequence-specific inhibitors of messenger RNA (mRNA) by binding to partially complementary regions in target mRNAs. Thus, the effectiveness of this post-transcriptional regulation of target genes depends on a complementarity between microRNA and mRNA. It is noteworthy that multiple microRNAs may cooperate in regulation of the same gene target and, conversely, any mRNA may contain multiple binding sites that are recognized by various miRNAs. Although the most common site for microRNA binding to mRNA is the 3′ UTR regions of target messenger RNA, in some cases, such as when mi122 interacts with hepatitis C virus (HCV), the binding site is the 5′UTR noncoding region of HCV RNA genome [23]. However, regardless of the place of interaction, microRNAs primarily act as mRNA-sequence-specific inhibitors of translation by the induction of mRNA degradation or simply translational repression. MicroRNAs are transcribed by RNA polymerase II that produce a long primary miRNA gene transcript (pri-miRNA). The pri-miRNAs then undergo sequential processing by nuclear and cytoplasmic enzymes. First, these RNAs are cleaved by the catalytic activity of DROSHA and DGCR8 microprocessor complexes into precursor miRNA (pre-miRNA) [24]. After exporting to cytoplasm, pre-miRNA is processed by the RNAse III enzyme Dicer to generate 18–25-nt duplex. The mature miRNA interacts with Argonaute protein and forms a miRNA-induced silencing complex ready for mRNA degradation and translation repression. Many microRNAs are key regulators of lipid metabolism [25]. The indispensable role in the regulation of cholesterol homeostasis has been indicated for miR-33 [21], miR-122 [26], mir-125b [27], miR-148a [28], mir-483 [29] and many others [30,31]. Among hundreds of microRNAs, miRNA-33 has been identified as a major regulator of cholesterol homeostasis and atherosclerosis [18,21]. Both members of the miR-33 family—miR-33a and miR-33b (the second being present only in primates)—are located in intron 16 of SREBP-2 and intron 17 of SREBP-1 gene, respectively and are controlled simultaneously with the SREBP genes [18] to govern cholesterol homeostasis. It was demonstrated that miR-33a and miR-33b are co-transcribed with SREBPF1 and SREBP2 and act to repress genes that oppose SREBP functions. For example, in the case of a low cholesterol concentration in a cell, SREB transcription factors and miR-33 are activated and cause an increase of cholesterol synthesis and, due to ABCA1 repression, the reduction of cholesterol efflux. The general regulator of several microRNAs involved in cholesterol metabolism, including miR-33, is a long noncoding primate-specific RNA (CHROME) expressed in macrophages and hepatocytes [32]. Elevated levels of CHROME in the plasma and atherosclerotic plaque were found in patients with coronary artery disease. It was observed that the level of CHROME expression depends on the levels of dietary and cellular cholesterol. CHROME becomes unregulated in response to cholesterol overload via LXR. Due to repression of miR-33 expression, CHROME post-transcriptionally regulates ABCA1 expression and increases cholesterol efflux. In turn, cells lacking CHROME exhibit a reduced expression of ABCA1 as a result of increased expression of miR-33. More details on the role of miR-33 in the regulation of cholesterol balance by influencing RCT and HDL biogenesis are presented in the following sections.

## 3. HDL Formation and microRNA-33 Influence

As the name indicates, high-density lipoproteins are a class of lipoproteins characterized by high density ranges from 1.063 to 1.25 g/mL. This heterogeneity is connected with different contents of apolipoproteins, enzymes and lipids in individual HDL subclasses (HDL2, HDL3 and very high-density HDL). HDL particles consist of divers proportions of phospholipids (PL), free cholesterol (FC), cholesterol esters (CE) and triacylglycerols (TG). Apo-A1 is a major structural protein accounting for approximately 70% of HDL proteins; however, other apolipoproteins, including A-II, A-IV, C-1I, C-II, C-III and E, are also associated with HDL [4]. Continuous remodeling of HDL is responsible not only for changing HDL density and shape but also size, ranging from 7.5 to 15 nm. The main function of HDL is reverse cholesterol transport from peripheral tissues, including macrophages in the vessel walls, to the liver, by which HDL may be anti-atherogenic. The negative correlation between HDL concentration and the risk of cardiovascular diseases strongly suggests cardioprotective properties of HDL [9]. These properties include, beyond the role of HDL in RCT, anti-inflammatory, anti-oxidative, anti-thrombotic and anti-apoptotic effects. Apo-A1, due to the interaction with ATP-binding cassette proteins, SR-B1 receptor and the activation of lecithin:cholesterol acyltransferase (LCAT), has an impact on both HDL biogenesis and reverse cholesterol transport [33]. The first step of HDL formation involves synthesis of Apo-A1 by the liver and intestine. The Apo-A1 mRNA and protein expression was recently demonstrated in samples of kidney renal clear cell carcinoma and in normal renal cells [34], but so far it has been difficult to assess to what extent the expression of Apo-A1 in the kidneys has an effect on the formation of HDL and on RCT. The formation of HDL particles that starts from Apo-A1 production depends on several factors. First, Apo-A1, newly-synthetized in hepatocytes and enterocytes, acquires phospholipid and cholesterol. The ABCA1 is critical for cholesterol and phospholipid efflux from these cells to lipid-poor Apo A1 and accounts for the formation of majority of nascent HDL particles [35]. Additionally, the phospholipid transfer protein (PLTP) promotes phospholipid efflux to HDL from peripheral cells and facilitates the movement of phospholipids between lipoproteins. The expression of ABCA1 is regulated by LXR family and retinoic acid receptors (RXR). When the cellular level of sterols increases, cholesterol is oxygenated to oxysterols that activate ABCA1 expression via activation of LXR and RXR. As a consequence, increased cholesterol efflux from cells is observed. It was demonstrated that mice with targeted knock-out of ABCA1 had a significantly reduced ABCA1 level in both the liver and intestine [36,37]. It was found that ABCA1 expression in the liver determines the HDL level in sera and influences the general cholesterol homeostasis [36]. It is important to note that ABCA1 mRNA with specific long 3′ UTR is highly susceptible to impact of micro RNA. The studies confirmed the mir-33-dependent repression of ABCA1 not only in hepatocytes but also in endothelial cells and macrophages [11,38]. MiR-33, like several other microRNAs, is regulated by CHROME [32], which influences the cholesterol efflux and HDL biogenesis. The knockdown of CHROME in human hepatocytes and macrophages increases the levels of miR-33 expression, and a result of this is reduced expression of ABCA1, which inhibits cholesterol efflux and HDL particle formation [32].

## 4. The Impact of microRNA-33 on Reverse Cholesterol Transport

The main part of reverse cholesterol transport is the transfer of cholesterol from peripheral cells, including foam cells in atherosclerotic plaques, to the liver. Peripheral cells accumulate cholesterol through the uptake of cholesterol-rich lipoproteins and de novo synthesis. In the human body, the excess of cholesterol is not simply degraded to acetyl-CoA, as it is in fatty acids. Thus, the only way to remove cholesterol is via the bile acid (BA) synthesis in the liver and BA excretion as bile-related products into feces. In this context, the effectiveness of RCT largely determines the balance between the absorbed cholesterol and the blood cholesterol. RCT begins with the hydrolysis of cholesteryl esters associated with cytoplasmic lipid-droplets by specific hydrolases and lipases [39]. In the early stage of RCT, cholesterol efflux is determined by cholesterol-deficient and phospholipid-depleted apoA-1 particles that are produced by the liver and intestine, as was described in the previous section. The nascent HDL particles acquire cholesterol and phospholipids by ABCA1, indicating the crucial role of ABCA1 expression not only for HDL formation but also for RCT [40]. Additionally, through the lecithin-cholesterol acyltransferase (LCAT) activity, cholesterol in HDL particles is esterified. Cholesteryl esters are moved to the core of HDL particles, forming a steady gradient of free cholesterol and enabling HDLs to accept more cholesterol [41]. Mature HDL particles can acquire additional cholesterol not only by ABCA1 but also via ATP-binding membrane cassette transport protein G1 (ABCG1) and scavenger receptor type B1 (SR-B1). Several studies have confirmed the contribution of the mature HDL particles in mediation of cholesterol efflux to HDL in macrophage foam cells [42,43,44]. By participating in this essential part of RCT, they contribute to the protection against atherosclerosis [45]. It was observed that overexpression of miR-33 down-regulates ABCA1 and ABCG1 transporters, thus decreasing HDL concentration in plasma. In this context, the impact of miR-33 on the expression of ATP-binding cassette transporters seems to be extremely important, decreasing cholesterol efflux from the atherosclerotic plague to circulating HDL particles. Moreover, it was established that microRNA-33 is responsible for remodeling of membrane microdomains and regulation of the innate immune response through the ABCA1 and ABCG1-dependent mechanism [46], indicating a close connection between cholesterol homeostasis and the immune system. This is another example confirming the importance of maintaining cholesterol homeostasis in the body. In continuing to describe the RCT process, the well-established role of SR-B1 in the selective uptake of cholesteryl esters from mature HDL to the liver should be emphasized. SR-B1, known to modulate HDL metabolism, is a glycoprotein of 509 aa that is involved in bidirectional cholesterol transport at the cell membrane [42]. SR-BI is expressed in the liver and tissues that need cholesterol for steroid synthesis. Unlike cholesterol, Apo-A1, the main protein component of HDL, is catabolized mostly by the kidneys and partially by the liver. Free Apo-A1 and lipid-poor Apo-A1 are filtered in the kidneys and taken up by the renal tubules [4]. The rate of Apo-A1 degradation depends on the degree of lipidation. No less important for the success of reverse cholesterol transport is the reciprocal exchange of cholesteryl ester for triglycerides. This process is mediated by cholesteryl ester transfer protein (CETP). The highest expression of CETP was found in the liver and in adipose tissue. CETP moves the bulk of the cholesteryl esters from HDL to apoB-containing lipoproteins (VLDL, IDL, and LDL). CETP is also responsible for the CE transfer among HDL subclasses. A study [47] has demonstrated that CETP inhibitors could have a significant impact on the management of dyslipidemic coronary heart disease patients. Both free and esterified cholesterol content of apoB-containing lipoproteins are taken up by the liver, predominantly via the low-density lipoprotein receptor (LDLR). In turn, HDL becomes enriched with triglycerides that are then degraded by hepatic lipase forming smaller HDL particles that are recognized by scavenger receptor type B1. Further catabolism of HDL-derived cholesterol occurs in the liver trough conversion of cholesterol into bile components. Improvement of RCT by upregulating of ABCA1 remains one of the potential targets for the development of new therapeutic agents against atherosclerosis. As miR-33 represses expression of ABCA1 transporter, antagonizing miR-33 seems to be an effective strategy for elevation of HDL in blood and for protecting patients from atherosclerosis. The results of many studies demonstrate that silencing of miR-33 in mice, with a variety of methods like targeted deletion [17], anti-sense nucleotides [18,19] or viral delivery inhibitors [21,38], significantly increased ABCA1 expression and the level of HDL in blood. The adverse effect of long-term silencing of miR-33, the increased level of triglyceride, was observed, but only in high-fat-diet mice [48]. In turn, another study [49] of the impact of hepatic miR-33 deficiency in mice demonstrated that loss of hepatic miR-33 improves metabolic homeostasis without any adverse effects. The absence of miR-33b in mice somehow limited the significance of these findings. Thus, a study performed in non-human primates (African green monkeys) with inhibiting of both miR-33a and miR-33b confirmed that inhibition of miR-33 not only increases the concentration of plasma HDL but also reduces VLDL triglycerides, in a model highly related to humans [50]. These results suggest the potential utility of miR-33 inhibition as a novel approach for the maintenance of cholesterol homeostasis.

## 5. Bile Formation and Secretion Can Be Deregulated by microRNA-33

Modulation of cholesterol metabolism in the human body depends on complex conditions encompassing both anabolic and catabolic processes. As excess cholesterol is not simply degraded, the only catabolic pathway responsible for cleansing the body of excess cholesterol is to convert it in the liver to bile acids (BAs) that are excreted into feces. The mixture of bile acids, cholesterol, phospholipids and ions forms so-called bile. The main functions of bile are the removal of metabolic wastes and the emulsification of dietary lipids. Deregulations of BA transportation as well as the alterations in BA receptor signaling are connected with development of dyslipidemias and atherosclerosis [51]. Thus, BA synthesis and secretion is the next crucial step of RCT that influences the whole-body cholesterol homeostasis. The bile acids level is transcriptionally controlled by the Farnesoid X receptor (FXR) [52]. FXR regulates bile acid synthesis depending on the accumulation of BAs [53]. Cholesterol delivered to the liver via LDLR and SR-BI can be eliminated by two pathways. Firstly, part of cholesterol is directly secreted into bile. Secondly, cholesterol is converted in hepatocytes into bile acids by the catalytic activity of cholesterol 7 alpha-hydroxylase (CYP7A1) [54]. As demonstrated in [55], the enzymatic activity of CYP7A1 can be inhibited by miR-33. After formation, bile acids are secreted across the apical membrane of hepatocytes by the canalicular transporters into the intestine. The most important in this step are transmembrane transporters, ATP-binding cassette: ABCB11, ABCG5, ABCB4, ATP8B1. Mutations that can inactivate the functions of ATP8B1, ABCB11, and ABCB4 are strictly connected with progressive familial intrahepatic cholestasis [56]. In the case of mutations in ABCG5 and ABCG8, the development of sitosterolemia is observed instead [57]. Interestingly, at this step of RCT a detrimental role of miR-33 was also identified. It was found that 3′ UTR of genes coding for ABCB11 and ATP8B1 revealed conserved parts complementary to miR-33 [58]. An experimental study [58] confirmed that both bile salt exporter ABCB11 as well as ATP8B1 are direct targets of miR-33. In normal conditions, both the ABC11 and ATP8B1 transporters promote hepatic clearance, directly via biliary lipid secretion and indirectly via arrangement of adequate canalicular membrane phospholipid asymmetry required for bile salt movement, respectively. Hepatic overexpression of miR-33 causes a significant reduction of ABCB11 and ATP8B1. As an effect of miR-33 overexpression, a decrease of biliary output and thus inhibition of cholesterol elimination in fecal bile acids was observed.

## 6. Conclusions

This review has been intended to demonstrate the impact of miR-33 on cholesterol homeostasis, and hopefully it has provided a positive answer to the titular question. miR-33 is one of the essential regulatory elements necessary to maintain a balance between dietary and blood cholesterol. This is primarily a result of its critical impact on the reverse transport of cholesterol. miR-33 controls cholesterol metabolism mostly by repressing ABCA1 and ABCG1, responsible for cholesterol efflux. As a result of balanced regulation by miR-33 and SREBP, we can observe a reduction of de novo cholesterol synthesis and an increase of cholesterol efflux from cells in the case of higher dietary intake of cholesterol and an increase of sterol concentration in cells afterwards. In turn, a decrease of cholesterol content in cells is sufficient to increase cholesterol synthesis and to abrogate cholesterol efflux. Besides the repression of ABCA1 and ABCG1, miR-33 influences other steps of RCT by targeting the transformation of cholesterol into bile salts and their transport. By controlling this final step of cholesterol elimination, miR-33 once again influences the homeostasis of cholesterol in an organism. In this context, the possibility of therapeutic use of miR-33 deserves special attention. The most common causes of cardiometabolic diseases are disorders of cholesterol metabolism, associated with an imbalance between cholesterol intake and elimination [59]. The World Health Organization estimates that the annual number of deaths from cardiovascular diseases, including atherosclerosis, will increase to over 22 million by 2030. While LDL levels are directly associated with atherosclerosis, HDL levels and the reverse cholesterol pathway show a strong inverse correlation with cardiovascular diseases. A majority of studies have demonstrated that antagonism of miR-33 in vivo increases circulating HDL and reverse cholesterol transport, thereby reducing the progression and enhancing the regression of atherosclerosis. Taking into account the number of genes that are targeted by mir-33, it can be assumed that miR-33 is a promising target for the treatment of dyslipidemia. A better understanding of the mechanisms that underlie miR-33 mediated regulation of cholesterol homeostasis may improve future therapies in the field.

## Data Availability

Not applicable.
